# Reputation in the Iterated Prisoner’s Dilemma: A Simple, Analytically Solvable Agents’ Model

**DOI:** 10.3390/e27060639

**Published:** 2025-06-15

**Authors:** Michał Cieśla

**Affiliations:** Institute of Theoretical Physics and Mark Kac Center for Complex Systems Research, Jagiellonian University, Łojasiewicza 11, 30-348 Kraków, Poland; michal.ciesla@uj.edu.pl

**Keywords:** iterated prisoner’s dilemma, reputation, cooperation and defection, agent model

## Abstract

This study introduces a simple model, which can be used to examine the influence of reputation on expected income achieved within the Iterated Prisoner’s Dilemma (IPD) game framework. The research explores how different reputation distributions among society members impact overall outcomes by modeling a society of agents, each characterized by a reputation score that dictates their likelihood of cooperation. Due to the simplicity of the model, we can analytically determine the expected incomes based on the distribution of agents’ reputations and model parameters. The results show that a higher reputation generally leads to greater expected income, thereby promoting cooperation over defection. However, in some cases, where there are more defecting individuals, the expected income reaches the maximum for agents with an average reputation, and then decreases for individuals who cooperate more. Various scenarios, including uniform, increasing, and decreasing reputation distributions, are analyzed to understand their effects on the promoted interaction strategy. Finally, we outline future extensions of the model and potential research directions, including the exploration of alternative reputation distributions, variable interaction parameters, and different payoff structures in the dilemma games.

## 1. Introduction

The Prisoner’s Dilemma (PD) is a simple two-player game in which each player must choose between two available strategies: cooperate (C) or defect (D) [1,2]. When both players cooperate, each receives a reward of *R*. If both of them are defective, each receives a punishment of *P*. If one player cooperates while the other defects, the cooperating player receives the sucker’s payoff *S*, while the defecting player receives the temptation payoff *T*. Typically, T>R>P>S and 2R>T+S. For example, T=5, R=3, P=1, and S=0 [2]. These rules can be written in the form of a payoff matrix:

(1) $$\begin{array}{c} \begin{array}{cc} \; & C\;D \end{array}\\ \begin{array}{cc} C & R\;S\\ D & T\;P \end{array} \end{array}\;⟶\;\begin{array}{c} \begin{array}{cc} \; & C\;D \end{array}\\ \begin{array}{cc} C & \;3\;0\\ D & \;5\;1 \end{array} \end{array}$$

When PD is played only once, a rational player should defect, as it always yields a higher payoff than cooperation, regardless of what the other player does. Therefore, the strategy when both players defect (D,D) corresponds to PD’s Nash equilibrium [3]. It remains unchanged when the game is repeated a definite number of times. In the last game, the optimal strategy for a rational player is to defect. As a result of the last game, both players are aware that the penultimate game becomes the last game, which should be taken into consideration. Therefore, using backward induction, one can prove that the Nash equilibrium corresponds to a (D,D) strategy for each game in a sequence. However, this strategy is not optimal, as both players could receive a higher reward of R>P if they cooperate. Therefore, especially when the number of consecutive games between the same two players is unknown, for example, if the probability of a repeated interaction with the same player is high enough, the optimal strategy can change to (C,C).

In general, such an iterated version of the Prisoner’s Dilemma (IPD) is one of the toy models in game theory actively used to study the phenomenon of cooperation, detection, and reputation [4,5,6,7,8,9,10,11,12,13,14]. For example, Cooper et al. explored the mechanisms behind cooperative behavior in PD games, particularly in settings where reputation effects are minimized or eliminated, and argued that a combination of altruism and limited reputation effects may better explain observed behavior [5]. However, numerous studies indicate that reputation is a crucial factor in strengthening cooperation [10,11,15,16,17,18,19]. Second-order reputation evaluation, where an individual’s reputation is updated based on their strategy and the reputations of their neighbors, has been found to enhance cooperation beyond spatial reciprocity [15]. The value of reputation is demonstrated by its impact on expected future payoffs and its tradability in reputation markets [16]. Experiments with random-matching PD games that incorporate information about opponents’ past actions and previous opponents’ behaviors have shown significant improvements in cooperation, especially when subjects have experienced low cooperation in no-information games [17]. These findings have important implications for the design and implementation of reputation systems in various contexts, including e-commerce and credit reporting [16,17].

Interestingly, in a spatial version of IPD, when cooperating or defecting players interact with their closest neighbors, clusters of cooperating agents can form [20,21]. Similar behavior was observed for humans playing the IPD game [22].

It is worth stressing that the type of interaction described in the Prisoner’s Dilemma game is observed in real life [1,9]. The IPD framework is also used to study trust and conflict resolution in long-term relationships [23]. It has been applied in disciplines such as economics, where businesses decide whether to compete or collaborate over repeated interactions [2,24], and in international relations, where countries develop policies of cooperation or retaliation based on others’ past actions, such as in arms control agreements or trade negotiations [25]. In evolutionary biology, IPD models how cooperative behavior can emerge and stabilize within populations over time, even when short-term incentives favor selfish behavior [23,26]. A key insight from the influential study by Axelrod and Hamilton was that simple strategies such as Tit-for-Tat can promote robust cooperation, even in environments where defection seems advantageous individually [2,25].

In this study, the PD game models the bilateral trading within a society of agents [27,28,29]. The general idea is based on assumptions taken from [29], where a randomly selected agent chooses a business partner based on the wealth of the partner. However, here, the second agent is chosen based on its reputation, which reflects its ability to cooperate rather than defect. It is based on the observation that in a modern world with almost no barriers to spreading information, reputation, along with price, is the most important factor when making trading arrangements [30,31]. Thus, agents who cooperate will be chosen more frequently as transaction parties than those who defect [6,32]. The main objective of this study is to propose a simple model that takes into account the most important factors in bilateral trading and can also lead to nontrivial conclusions. The study of the proposed model focuses on determining how the expected income of a member of such a society depends on their reputation, measured in terms of the probability of cooperation.

This paper is organized as follows: Section 2 describes how the trading agents are chosen and what the results of their interaction are. In Section 3, the expected income dependence on the reputation of the agents is derived for several different distributions of the reputation of the agents in the society. The results obtained are discussed in Section 4, after which the study is concluded.

## 2. Model

The model is based on two assumptions. First, each agent can play the PD game, and second, they have a limited opportunity to choose an opponent. They want to play with one who cooperates the most. To formulate it, let us assume that there are N≫1 independent agents. Each agent is characterized by a single parameter *q*, which represents the agent’s reputation. This reputation is defined here as the probability that the agent will cooperate with another agent during a game, regardless of the other agent’s reputation. Two interacting agents are selected from the entire population using the following rules:

- The first agent is chosen randomly with uniform probability. Thus, the probability of selecting a given agent is $P_{1}=1/N$, and it does not depend on the reputation of the agent.
- The second agent is selected in a two-stage process. Firstly, n<N agents are randomly selected according to a uniform probability (the probability of selecting a given agent is n/(N−1)). Then, from this group of *n* agents, one with the highest reputation is taken.

This type of matching is often referred to as a selective assortment [33,34], however, there is no classical setup when agents are divided into two groups: always cooperating and always defecting [35]. Here, most players can use both strategies simultaneously, albeit with different probabilities, which seems to be closer to reality. The probability that the highest reputation $q_{max}$ among the group of *n* agents is smaller than a given value of *x* is

(2) $$Prob\left(q_{max}<x\right)=Prob\left(q_{1}<x\right)\cdot Prob\left(q_{2}<x\right)\cdot ...\cdot Prob\left(q_{n}<x\right)={\left[Prob\left(q<x\right)\right]}^{n}.$$

Note that the above equation uses the cumulative distribution functions, and thus, the probability density function of the second agent’s reputation is

(3) $$p_{q_{max}}\left(x\right)=\frac{d}{dx}Prob\left(q_{max}<x\right)=n{\left[Prob\left(q<x\right)\right]}^{n-1}p_{q}\left(x\right),$$

where $p_{q}\left(x\right)$ is the probability density function of finding an agent of reputation *q* in the agents’ society.

Two selected agents interact with each other and choose their strategy (cooperate or defect) randomly and independently, based on their reputation. Thus, the result of the game for the first agent is

(4) $$G_{1}\left(q_{1},q_{2}\right)=q_{1}\left[q_{2}R+\left(1-q_{2}\right)S\right]+\left(1-q_{1}\right)\left[q_{2}T+\left(1-q_{2}\right)P\right].$$

Analogously, $G_{2}\left(q_{1},q_{2}\right)=G_{1}\left(q_{2},q_{1}\right)$. Each agent collects their gain from this game, and the entire process repeats: two agents are selected, they interact, gain the prize, and the process continues. For convenience and without loss of generality, let us assume that there are *N* such iterations. It is also assumed that the agents’ reputations remain unchanged during the whole process, which differs from other approaches, where agents’ reputations are adjusted to their former games [36].

## 3. Results

The expected income of the first selected agent of reputation *q* is

(5) $$I_{1}\left(q\right)=\int_{0}^{1}p_{q_{max}}\left(x\right)G_{1}\left(q,x\right)\;dx,$$

because his partner’s reputation is given by the $p_{q_{max}}\left(x\right)$ probability density function. On the other hand, the expected gain of the second agent is

(6) $$I_{2}\left(q\right)=\int_{0}^{1}p_{q}\left(x\right)G_{1}\left(q,x\right)\;dx.$$

Thus, the total expected income of a given agent after *N* iterations is

(7) $$I\left(q\right)=N\left[\frac{1}{N}I_{1}\left(q\right)+\frac{n}{N-1}{\left(\int_{0}^{q}p_{q}\left(x\right)dx\right)}^{n-1}I_{2}\left(q\right)\right],$$

where the two components relate to the possibility of being the first or the second chosen agent, respectively. The prefactors before $I_{1}\left(q\right)$ and $I_{2}\left(q\right)$ correspond to the probabilities that the agent will be selected as a first and second player, respectively. The integral in the second component corresponds to the probability that the reputation of the second agent is the highest among the group of *n* chosen agents—see (2).

In the limit of large *N*, the expected income of a given agent (7) depends only on his reputation and parameters of the model, which here are *n*—the size of the group of agents from which we select the one with highest reputation, and $p_{q}\left(x\right)$—the reputation probability density function. Because we do not know which distribution of $p_{q}\left(x\right)$ resembles reality the best, we will analyze the model output for several typical example forms of $p_{q}\left(x\right)$, namely, the uniform distribution, increasing and decreasing distributions—where more players are of higher or lower reputation, respectively. Next, we look at a distribution condensed around q=0.5, and one that promotes simultaneously agents of very high and very low reputation.

### 3.1. Case 1: $p_{q}\left(x\right)=Const$

The first studied case is of a reputation uniformly distributed in the society of the agents:

(8) $$p_{q}\left(x\right)=\left\{\begin{array}{cc} 1 & x\in [0,1]\\ 0 & \mathrm{otherwise} \end{array}\right.\;.$$

Here, the expected income can be calculated analytically:

(9) $$\begin{array}{ccc} I(q) & = & \frac{1}{2}nq^{n-1}\left(-Pq+P+qR+qS-qT+T\right) \end{array}$$

(10) $$\begin{array}{ccc} & & +\frac{nqR-nqT+nT-Pq+P+qS}{n+1}, \end{array}$$

which, for standard PD parameters T=5, R=3, P=1 and S=0, reduces to

(11) $$I\left(q\right)=\frac{1}{2}n\left(6-3q\right)q^{n-1}+\frac{-2nq+5n-q+1}{n+1}.$$

**Figure 1:**
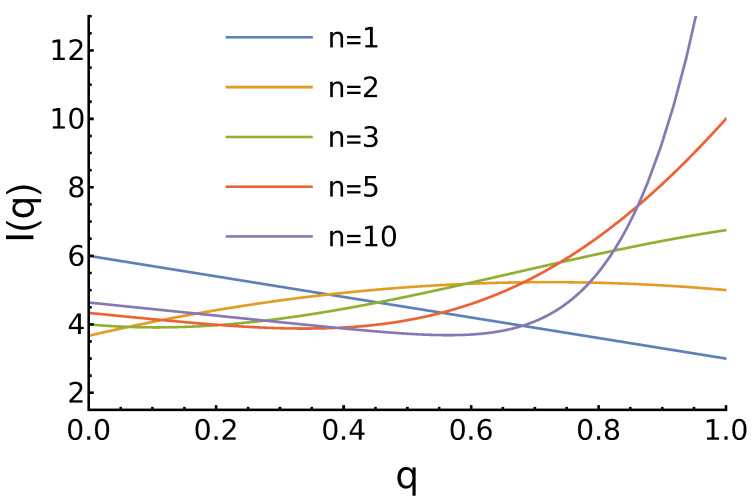
Dependence of the agents’ expected income (7) on its reputation for uniform reputation distribution and different values of the parameter *n*. For n=1, the income decreases linearly with increased *q*. For n=2, a global maximum at average *q* is observed. For larger *n*, we observe a local minimum and then the largest income at q=1.

The character of income dependence on *q* strongly depends on the parameter *n*. For example, for n=1, $I_{n=1}\left(q\right)=6-3q$, and its maximum corresponds to q=0, which is in accordance with the Nash equilibrium of the PD game. For n=2, $I_{n=2}\left(q\right)=\frac{1}{3}\left(-9q^{2}+13q+11\right)$, and the “always defect” strategy becomes the worst. The expected income has a maximum at $q=\frac{13}{18}\approx 0.722$. For n=3, $I_{n=3}\left(q\right)=-\frac{9}{2}q^{3}+9q^{2}-\frac{7}{4}q+4$, and here we see a global minimum for $q=\frac{1}{18}\left(12-\sqrt{102}\right)\approx 0.106$, but the maximum income is achieved by the most reputable agent: I(q=1)=6.75. For higher *n*, the character of I(q) does not change qualitatively. As *n* increases, the minimum shifts to the right, and the maximum at q=1 increases. These and other results are presented in Figure 1.

### 3.2. Case 2: $p_{q}\left(x\right)∼x$

Now, assume that the probability of finding an individual with a given reputation increases linearly with it. For example,

(12) $$p_{q}\left(x\right)=\left\{\begin{array}{cc} 2x & x\in [0,1]\\ 0 & \mathrm{otherwise} \end{array}\right.\;,$$

which leads to

(13) $$\begin{array}{ccc} I(q) & = & \frac{1}{3}nq^{2(n-1)}\left(-Pq+P+2qR+qS-2qT+2T\right) \end{array}$$

(14) $$\begin{array}{ccc} & & +\frac{2nqR-2nqT+2nT-Pq+P+qS}{2n+1}. \end{array}$$

For T=5,R=3,P=1 and S=0 the above relation reduces to

(15) $$I\left(q\right)=\frac{1}{3}n\left(11-5q\right)q^{2(n-1)}+\frac{-4nq+10n-q+1}{2n+1}.$$

**Figure 2:**
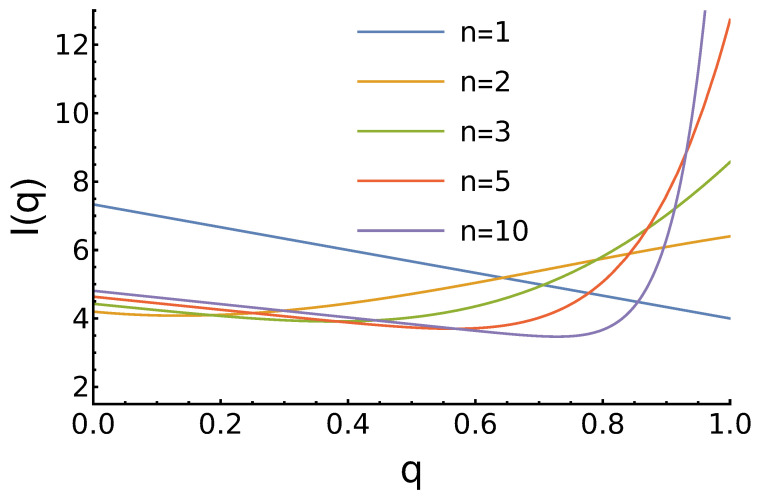
Dependence of the agents’ expected income (7) on its reputation for growing reputation distribution and different values of the parameter *n*. For n=1, the income decreases linearly with increased *q*. For larger *n*, we observe a local minimum and then the largest income at q=1.

As in the previous case, the expected income depends on the parameter *n*. These dependencies are shown in Figure 2.

Although there are not many qualitative differences with the previous case of constant reputation distribution $p_{q}\left(x\right)$, it is worth noting the following:

- For n=1, the maximum for q=0 is higher as there are more opponents, who prefer to cooperate;
- For n=2, there is no maximum for medium *q*. In contrast, we observe the minimum near q=0.134. The position of this minimum shifts to the right as *n* increases;
- For n≥2, the maximum is at q=1 and it grows with the increase of *n*.

### 3.3. Case 3: $p_{q}\left(x\right)$ Decreases with an Increase of *x*

An opposite case is when the majority of agents have a poor reputation. Here, let us consider two possibilities. In the first one, the decrease is given by a linear function

(16) $$p_{q}\left(x\right)=\left\{\begin{array}{cc} 2-2x & x\in [0,1]\\ 0 & \mathrm{otherwise} \end{array}\right.\;,$$

and in the second one, the decrease is exponential

(17) $$p_{q}\left(x\right)=\frac{a\exp(a)}{\exp(a)-a-1}\left\{\begin{array}{cc} \exp(-ax)-\exp(-a) & x\in [0,1]\\ 0 & \mathrm{otherwise} \end{array}\right.\;.$$

Note that the prefactor [aexp(a)]/[exp(a)−a−1] is to normalize the distribution, and the component exp(−a) is to ensure that $p_{q}\left(x=1\right)=0$. The parameter a>0 corresponds to the slope of the exponential decrease—the larger *a* is, the faster $p_{q}\left(x\right)$ decreases.

**Figure 3:**
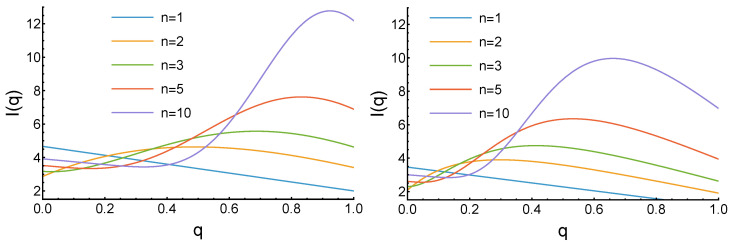
Dependence of the agents’ expected income (7) on its reputation for growing reputation distribution and different values of the parameter *n*. The left plot corresponds to the linear decrease of $p_{q}\left(x\right)$ and the right if for exponential decay with a=5. In both, for n=1, the income decreases linearly with increased *q*, and for larger *n*, a global maximum at average *q* is observed. The maximums shift to the right as *n* increases. The maximums are closer to the q=1 in the case of linear decrease (left panel), where there are fewer agents of very low reputation.

In the case of linear decrease, some of the integrals (5)–(7) that define the expected income for arbitrary *n* cannot be evaluated using only elementary functions. However, for specific *n* values, I(q) has a polynomial form. For exponential decrease, the general form of the expected income is complicated, but it can still be calculated analytically for specific values of parameters. The results are presented in Figure 3.

For n=1, as previously, the “always defect” (q=0) strategy is the best, but it is much less profitable due to the higher probability of selecting a defecting opponent: $I_{n=1}\left(0\right)\approx 4.667$ for the linear decay, and $I_{n=1}\left(0\right)\approx 3.460$ for exponential one. For n=2, we observe the maximum $I_{n=2}\left(0.489\right)\approx 4.634$ for the linear decay, and $I_{n=2}\left(0.297\right)\approx 3.897$ for exponential one. These maxima increase with the growth of the parameter *n* and also move in the direction of larger reputation *q*. For example, $I_{n=3}\left(0.687\right)\approx 5.571$, $I_{n=5}\left(0.831\right)\approx 7.624$, and $I_{n=10}\left(0.924\right)\approx 12.775$ for the linear decay, and $I_{n=3}\left(0.413\right)\approx 4.746$, $I_{n=5}\left(0.532\right)\approx 6.352$, and $I_{n=10}\left(0.661\right)\approx 9.965$ for exponential decay. In contrast to previous cases, all these maxima are not reached for the maximum reputation of q=1. It is worth mentioning that in the exponential case, there are fewer individuals with higher reputation than in the linear case. Thus, in general, the more defective players in the society, the lower the reputation, which guarantees the largest profit.

To complete the analysis, we study two more cases. The first is when the distribution of $p_{q}\left(x\right)$ has the maximum for q=0.5 and the minima for q∈{0,1}. The second, which is opposite to the first one, namely, $p_{q}\left(x\right)$, has a minimum of q=0.5 and maxima of q∈{0,1}.

### 3.4. Case 4: $p_{q}\left(x\right)$ with Maximum at $q=\frac{1}{2}$ and Minima for q∈{0,1}

To model this case, we chose the probability distribution function of reputation *q* defined as follows:

(18) $$p_{q}\left(x\right)=\left\{\begin{array}{cc} 6x(1-x) & x\in [0,1]\\ 0 & \mathrm{otherwise} \end{array}\right.\;.$$

**Figure 4:**
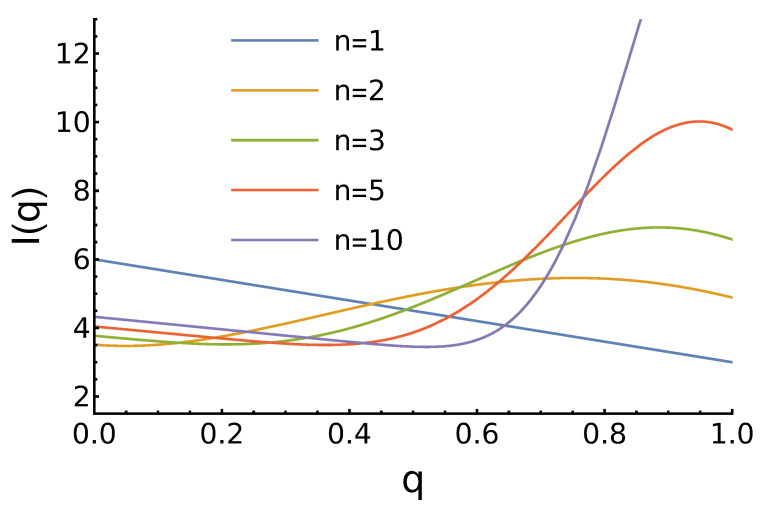
Dependence of the agents’ expected income (7) on its reputation for growing reputation distribution and different values of the parameter *n*. For n=1, the income decreases linearly with increased *q*. For larger *n*, a global maximum at q<1 is observed, similar to that in the left panel in Figure 3.

Similarly to the previous case, the total income for arbitrary *n* does not have a compact, elementary form, but for specific *n*, it is again given by a polynomial in reputation *q*. The results are shown in Figure 4.

Qualitatively, the results are similar to those from the previous case. The main difference is that the “always defect” strategy yields better results here, especially for n=1. For larger *n*, the maxima are higher and also closer to the q=1 limit.

### 3.5. Case 5: $p_{q}\left(x\right)$ with Minimum at $q=\frac{1}{2}$ and Maxima for q∈{0,1}

In the last studied case, we also use a quadratic function to model agents’ reputation distribution:

(19) $$p_{q}\left(x\right)=\left\{\begin{array}{cc} -12x(1-x)+3 & x\in [0,1]\\ 0 & \mathrm{otherwise} \end{array}\right.$$

**Figure 5:**
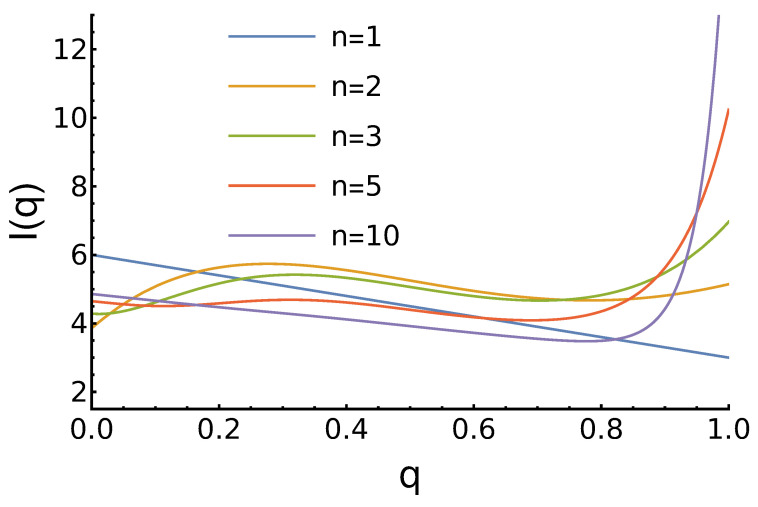
Dependence of the agents’ expected income (7) on its reputation for growing reputation distribution and different values of the parameter *n*. For n=1, the income decreases linearly with increased *q*. For larger *n*, we observe global (n=2) or local maxima for smaller *q*, then local minima for larger *q*. For n≥3, the income reaches its highest value at q=1.

Here, also, income I(q) has a polynomial form for specific values of the parameter *n*. The results are presented in Figure 5.

The results here are the most similar to those from Case 2, where the decreasing distribution of agents’ reputation was studied. However, it should be noted that for moderate values of *n*, we observe some fine structure of local maxima around q≈0.4 and minima near q≈0.8. Finally, for the studied models, we can estimate the expected agent’s income in the society

(20) $$E\left(I\right)=\int_{0}^{1}p_{q}\left(x\right)I\left(x\right)dx,$$

and its dispersion

(21) $$\sigma \left(I\right)=\sqrt{\int_{0}^{1}p_{q}\left(x\right){\left(I(x)-E[I]\right)}^{2}dx}.$$

The results are collected in Table 1.

The largest overall results are obtained for case 2, where cooperation is more probable. On the other hand, the lowest expected income is for case 3 with exponential probability decay, where most agents defect. Similarly, the dispersion is larger where more cooperating agents are present. Interestingly, if we treat dispersion as a measure of inequality, we see that more egalitarian societies tend to be poorer and contain a higher proportion of defecting members. Comparing the two other cases, the better results are for case 5, where, again, there are many agents with higher reputations. Thus, on average, the studied model fosters cooperation over defection, as is the case in the IPD game.

All notebooks with the results, calculations, and analytical formulas are attached as supplementary materials. The reader is encouraged to use them and experiment with the model’s results using different parameters.

## 4. Discussion

Despite the significant differences in the assumed reputation distribution among the agents’ society, all the results presented are qualitatively quite similar.

First, for n=1, the “always defect” strategy is the most profitable [7,8]. In this case, the reputation does not affect the number of possible interactions that could lead to more opportunities to gain a positive reward from the game. In *N* games, the player plays twice on average and obtains a temptation (T) or a punishment (P) payoff, depending on the opponent’s behavior. The strategy is more prominent where opponents have higher reputations, as the temptation (T) payoff occurs more often.

On the other hand, the “always defect” strategy is the worst for n=2. To better understand this, let us revisit the first case and the relation (7) that describes the income I(q). The “always defect” agent will play only if they are chosen as the first player, and the payoff from this game is

(22) $$I\left(q=0\right)=I_{1}\left(q=0\right)=\int_{0}^{1}p_{q}\left(x\right)\left[xT+(1-x)P\right]dx,$$

which, in the case of $p_{q}\left(x\right)=const$, T=5, and P=1, reduces to $I\left(q=0\right)=\frac{1+5n}{1+n}$, while for $p_{q}\left(x\right)∼x$ and the same *T* and *P*, to $I\left(q=0\right)=\frac{1+10n}{1+2n}$. Both of these relations grow with increasing *n*. This is because the larger the set of agents selected as potential opponents, the higher the expected reputation of the most reputable agent in this set. Thus, the temptation (T) payoff for the first, “always defect”, player becomes more probable than the punishment (P) one. Therefore, n=2 is the worst possible case for such a player.

For higher *n*, agents with a high reputation gain greater expected rewards. This is because their reputation often leads them to play more often as second-tier players. It is especially profitable when there are many reputable players with whom they can cooperate and receive a reward of *R*. Only in the third and fourth of the studied cases, where there are less reputable agents, is the “always cooperate” strategy not optimal, as opponents often defect, resulting in no payoff for the cooperating player. In such a case, the optimal reputation is lower than q=1, especially when most players are defective, as in case 3 with exponential decay of the probability of finding a cooperative opponent. Either way, the larger the parameter *n*, the higher the optimal reputation. On the other hand, for each studied case, the dependence of expected income on the player’s reputation is typically highly nonlinear and nonmonotonic, which agrees with the previous observation that the coexistence of cooperating and defective players may lead to changes in the Nash equilibrium [37].

## 5. Conclusions

We presented a simple model of a society of agents that interact with themselves based on the rules of the Prisoner’s Dilemma game. Agents can cooperate or defect according to their reputation attribute. We examined the model with six specific distributions of agents’ reputation in the society. The results obtained suggest that, despite significant differences in reputation distributions, all variants studied here have many common characteristics. For example, agents with a higher reputation generally reach the maximum expected income [38]. On the other hand, a medium reputation often gave worse incomes than the “always defect” strategy.

Because the model can be solved analytically, it opens up various opportunities for future studies, ranging from other distributions of reputations to the introduction of the variable parameter *n* from one game to another. Other possible extensions can include fluctuations in the reputation distribution, interaction probabilities based on the distance between agents defined by a graph structure, or in a different manner [39]. Additionally, a player’s strategy can change depending on its location [40] or history of past games [41]. Moreover, instead of total income, one can study income distribution and wealth inequality [42]. Agents could also adjust their strategies to maximize the expected rewards [43]. Lastly, different Prisoner’s Dilemma game payoff tables can be tested, or even other dilemma games, e.g., the Snowdrift game or Stag Hunt game [21,44]. 

## Figures and Tables

**Table 1:** Expected income (20) and its dispersion (21) in brackets of the agents’ society for each studied case and several values of the parameter *n*.

	n=1	n=2	n=3	n=5	n=10
case 1	4.50 (0.87)	4.83 (0.45)	5.00 (0.94)	5.17 (1.67)	5.32 (2.86)
case 2	5.11 (0.79)	5.33 (0.71)	5.42 (1.44)	5.52 (2.40)	5.59 (3.94)
case 3 (linear)	3.78 (0.63)	4.09 (0.51)	4.27 (0.88)	4.47 (1.41)	4.70 (2.25)
case 3 (exponential)	3.03 (0.40)	3.25 (0.55)	3.41 (0.87)	3.60 (1.28)	3.87 (1.89)
case 4	4.50 (0.67)	4.76 (0.63)	4.89 (1.19)	5.02 (1.95)	5.16 (3.16)
case 5	4.50 (1.16)	4.95 (0.43)	5.14 (0.71)	5.32 (1.48)	5.43 (2.76)

## Data Availability

The original contributions presented in this study are included in the supplementary material. Further inquiries can be directed to the corresponding author.

## Supplementary Materials

The following supporting information can be downloaded at: https://www.mdpi.com/article/10.3390/e27060639/s1.

## Abbreviations

The following abbreviations are used in this manuscript:

PD	Prisoner’s Dilemma
IPD	Iterated Prisoner’s Dilemma
